# Unique Honey Bee (*Apis mellifera*) Hive Component-Based Communities as Detected by a Hybrid of Phospholipid Fatty-Acid and Fatty-Acid Methyl Ester Analyses

**DOI:** 10.1371/journal.pone.0121697

**Published:** 2015-04-07

**Authors:** Kirk J. Grubbs, Jarrod J. Scott, Kevin J. Budsberg, Harry Read, Teri C. Balser, Cameron R. Currie

**Affiliations:** 1 Department of Biology, University of North Carolina, Chapel Hill, North Carolina, United States of America; 2 Bigelow Laboratory for Ocean Sciences, East Boothbay, Maine, United States of America; 3 Department of Bacteriology, University of Wisconsin, Madison, Wisconsin, United States of America; 4 Department of Soil and Water Science, University of Florida, Gainesville, Florida, United States of America; National Research Laboratory of Defense Proteins, REPUBLIC OF KOREA

## Abstract

Microbial communities (microbiomes) are associated with almost all metazoans, including the honey bee *Apis mellifera*. Honey bees are social insects, maintaining complex hive systems composed of a variety of integral components including bees, comb, propolis, honey, and stored pollen. Given that the different components within hives can be physically separated and are nutritionally variable, we hypothesize that unique microbial communities may occur within the different microenvironments of honey bee colonies. To explore this hypothesis and to provide further insights into the microbiome of honey bees, we use a hybrid of fatty acid methyl ester (FAME) and phospholipid-derived fatty acid (PLFA) analysis to produce broad, lipid-based microbial community profiles of stored pollen, adults, pupae, honey, empty comb, and propolis for 11 honey bee hives. Averaging component lipid profiles by hive, we show that, in decreasing order, lipid markers representing fungi, Gram-negative bacteria, and Gram-positive bacteria have the highest relative abundances within honey bee colonies. Our lipid profiles reveal the presence of viable microbial communities in each of the six hive components sampled, with overall microbial community richness varying from lowest to highest in honey, comb, pupae, pollen, adults and propolis, respectively. Finally, microbial community lipid profiles were more similar when compared by component than by hive, location, or sampling year. Specifically, we found that individual hive components typically exhibited several dominant lipids and that these dominant lipids differ between components. Principal component and two-way clustering analyses both support significant grouping of lipids by hive component. Our findings indicate that in addition to the microbial communities present in individual workers, honey bee hives have resident microbial communities associated with different colony components.

## Introduction

Most, if not all, metazoans are associated with consistent and sometimes specialized microbial communities, and these microbiomes are increasingly recognized for their important role in shaping the biology of their hosts [1]. Social insects allow us to explore interesting questions associated with metazoan microbiomes because they form colonies of related individuals that typically occur in specialized nests. Further, the nests of some social insects have spatial structuring based on the rearing of young and long term storage of nutrient rich material. In two recent studies of ants, the microbial communities associated with different castes and nest components were shown to vary more by component than by species [2,3]. These studies suggest that just as different body locations in mammals appear to have distinct resident microbiomes [4,5], specific microbial communities may be associated with the different components of social insect colonies.

Honey bees (*Apis mellifera*) maintain complex hives composed of different constituent parts. Hive membership includes three different castes of bees: male drones, up to ~80,000 sterile female workers, and (generally) a single egg-laying queen. The female workers practice a division of labor partly based on age, with younger individuals performing tasks within the hive while older workers engage in the ecologically and economically important task of plant pollination [6–8]. In addition to the complexity associated with a large and structured work force, hives of honey bees also contain honey, pollen, uncapped larvae, egg-containing cells, capped pupae-containing cells, and the wax comb that houses these components. These different components are typically spatially segregated within the hive, often occurring within individual cells within the comb. In addition, bees use propolis, a mixture of wax and tree resins, to seal and protect the hive from both micro- and macro-intruders [9,10].

A diverse array of microbes is associated with honey bees and their hives. Bees, especially the developing brood, are highly susceptible to pathogenic microbes. American foulbrood (*Paenibacillus larvae*) is one of the most widespread and destructive brood diseases [11,12]. The bacterial pathogen infects larvae that are less than three days old, germinating in the gut and eventually killing the larva. However, microbes are also believed to play beneficial roles within honey bee hives. The microbial community that exists within the digestive tracts of worker bees and helps with nutrient breakdown is composed of eight consistent bacterial phylotypes that vary in relative population sizes between individuals [13–20]. Moreover, bacteria and fungi are implicated in the fermentation of pollen to bee bread [21–26] and have been linked to ripening of honey [24,27]. The further investigation of honey bees, their digestive tracts, and bee bread has revealed that increased microbe diversity is linked with the overall genetic diversity of the worker population [28].

To generate an overall description of the microbiota of honey bee colonies, we sampled individual hive components, including workers, honey, comb, propolis, pollen stores and pupae from 11 *Apis mellifera* hives and employed a hybrid of fatty acid methyl ester (FAME) and phospholipid fatty acid (PLFA) analyses. In this method, lipid biomarkers are used to identify and provide relative abundance estimates for specific groups of microbes within a given niche as FAMEs are formed from all fatty acids and PLFAs are the constitutive lipids of cell membranes, both of which are unique to groups of organisms [29]. This approach is not subject to culture bias and measures only lipids from viable cells as PLFAs are quickly degraded after cell death [30,31], thus offering some advantages over other methods. However, lipid-based profiling is limited in that it only provides broad phylogenetic resolution. Through the use of this technique, we describe here the broad microbial community associated with honey bee hives. Further, we determine the relative abundance and lipid-based richness of viable microbes associated with stored pollen, adults, pupae, honey, empty comb, and propolis. Finally, we examine if different microbial communities are associated with different hive components in honey bees.

## Materials and Methods

### Ethics Statement

No human or protected animal subjects were used in this study. Five hives were located on land owned by and sampled with permission from Eugene Woller. Three hives were located on land owned by and sampled with permission from Craig Petros. The Currie Lab maintained two hives on land owned by the University of Wisconsin at Madison. One hive was maintained by the Currie Lab at the Henry Vilas Zoo with permission from Rick Bilkey.

### Sample Collection

In the midsummers and early falls of 2006, 2008 and 2010, honey bee hive materials were sampled from apiaries in South Central Wisconsin from a total of eleven outwardly healthy hives. Three hives were sampled in 2006, six were sampled in 2008, and two were sampled in 2010. No hives were sampled twice in order to ensure that each hive was unique and that similarities between two hives could not be explained by a single persistent colony or queen. Please refer to S1 Table for a full sample inventory including the details of the specific components obtained from each hive and the number of samples taken from each. 50 mL tubes were filled for each component sampling. All samples except propolis were obtained by removing brood frames from the hive and transporting them back to the laboratory in a sterile container. Once in the laboratory (after a maximum of an hour’s transit time), the brood frames were placed in a biosafety hood and sampled immediately. Pupae of all ages were dissected out of cells, pollen was cut out of sections of full comb containing only pollen, and adults were collected from around the hive and brood frames (making age estimates infeasible). Pupae and adults were not surface sterilized. Honey was collected by cutting out and disrupting sections of full honey comb. The comb was then placed in a sterile beaker while the honey collected in the bottom. The resulting honey was then transferred to sterile 50 mL falcon tubes (Becton Dickinson, Franklin Lakes, NJ) and centrifuged at 4100 rpm (IEC CL31 Multispeed, Thermo Scientific, Waltham, MA) for 10 minutes. Centrifugation caused the wax comb to collect on top of the honey and allowed for easy disposal of extra particulates. The empty comb was cut out of frames. Propolis was collected using a hive tool to scrape the inside of hives. The scrapings were kept in 50 mL falcon tubes and stored at -80°C upon return to the laboratory. All samples were collected with ethanol and flame sterilized utensils and stored in sterile 50 mL falcon tubes at -80°C within 8 hours of removing corresponding brood frames from hives.

### Lipid Extraction, Analysis, and Phylogenetic Assignment

Microbial communities were assessed with a hybrid phospholipid fatty acid (PLFA) and fatty acid methyl ester (FAME) method. The technique employed is based on the extraction of lipid biomarkers from the hive components. Samples were frozen at -20°C, lyophilized, and milled to #40 mesh size prior to PLFA extraction. Lipids were then extracted and purified using a modified Bligh and Dyer technique [32]; FAME analysis was implemented in accordance to the method described by Microbial ID Inc [33]. Briefly, lipids were extracted from lyophilized and milled hive components (Sample Masses: S1 Table) by shaking with two successive 1-h washes in a solution of 2.8 mL 0.1 M phosphate buffer (pH 7.5), 6 mL methanol, and 3 mL chloroform. Solids were separated from liquids by centrifugation. The phosphate buffer and chloroform were re-added to affect organic-aqueous phase development. The samples were then incubated at room temperature overnight for phase separation. The organic lipid-containing phase was separated and the solvent was evaporated using a RapidVap (LabConco, Kansas City, MO). MIDI company’s procedure for FAME was then affected by saponifying cells to cleave cellular lipids and generate fatty-acid sodium-salts, methylating fatty-acid sodium-salts to FAMEs, extracting FAMEs to an organic phase, and finally, purifying the extracted FAMEs with a base wash [33]. Purified FAMEs were suspended in a 1:1 solution of hexane and MTBE (Fisher Scientific, Pittsburgh, PA). This solution also contained two internal standards of known concentration: 9:0 (nonanoic methyl ester) and 19:0 (nonadeconoic methyl ester) (Sigma, St. Louis, MO). All tubes and caps were hexane-rinsed Teflon or glass baked at 550°C for 3-h in order to prevent contamination by non-relevant lipids.

A Hewlett-Packard 6890 Gas Chromatograph (Wilmington, DE) configured and maintained for lipid analysis according to the recommendations of MIDI [33] was used to analyze two microliters of FAME solution. Instrument parameters were specified and peaks were identified using MIDI’s (MIDI, Newark, DE) EUKARY method. Fatty acid concentration was assessed by comparing peak areas of the samples to that of the two internal standards, 9:0 (nonanoic methyl ester) and 19:0 (nonadeconoic methyl ester) (Sigma, St. Louis, MO), of known concentration. In all subsequent analyses, fatty acids that were at an average abundance of <0.5 mol% or present in <3 samples were excluded. Resulting lipid data (mol %) were normalized via arcsin-transformation.

In order to generate an understanding of which taxonomic groups may be represented in each component, the total set of detected lipids was cross-referenced with previous studies [30,34–45] (Table 1). In the case of a lipid that implicated several groups, the most general category was used (e.g., Fungi, Plants and Mammals would be Eukaryotes). When a lipid was specifically indicated in several non-informatively sortable groups, it was deemed to be non-specific and placed in the non-specific group. When a lipid was not found previously referenced, it was placed in the unknown group. Those lipids that are generally found in most domains of life were classified as widespread. Some detected lipids were unable to be specified beyond the possibility of two different lipids with identical masses. These were indicated as the two lipids separated by a “/”. In each of these cases, the software named the most likely lipid first. We then considered these to represent the specific lipid if one of the pair was always named first. Finally, some non-standard lipids were unidentifiable and are designated with an “Unk” prefix or non-standard nomenclature. All lipids were included in subsequent analysis unless noted. This method of phylogenetic assignment results in coarse resolution, but given the nature of the lipid analysis method used and that it has not been used with samples of this type before, a conservative approach is more appropriate.

**Table 1:** Set of Detected Lipids and Their Indications.

**Lipid**	**Indicates**	**Lipid**	**Indicates**
9:0	Bacteria: Non-specific	18:0	Bacteria: Non-specific
11:0-2OH	Gm-	10Me18:0	Actinomycetes
*i*11:0	Gm+	18:1(ω?)Alc	-
12:0	Bacteria: Non-specific	18:1ω5c	Gm-
12:0-3OH	Gm-, γ-proteo	18:1ω6c	Gm-
12:1ω8c	Gm-, Methanotrophs	18:1ω7c	Gm-, γ-proteo (EV), BC
14:0	Bacteria: Non-specific	11Me18:1ω7c	-
*i*14:0	Gm+, BC, Act	18:1ω9c	Fungi (SP or Ecto)
14:1ω11c	Gm-	18:2ω6,9c	Fungi (SP)
14:1ω7c	Gm-	18:3ω6,9,12c	Eukaryotes
14:2 ω6c/*a*14:0	Bacteria: Non-specific	19:0	Bacteria: Non-specific
15:0	Bacteria: Non-specific	10Me19:0	Actinomycetes
*a*15:0	Gm+, BC, Act	*cy*19:0	Gm-(An), Gm+, γ-proteo, BC
*i*15:0	Gm+, BC, Act	19:1(ω11?)Alc	-
*i*15:1	Gm+	*i*19:1	Gm+, *BC*
16:0	Widespread	19:1ω8t	Gm-
10Me16:0	Actinomycetes	20:0	Eukaryotic
*i*16:0	Gm+, BC, Act	*i* 20:0	Gm+
*i*16:1	Gm+, BC	20:1ω5c	Gm-
16:1ω5c	Fungi (AMF)	20:1ω6c	Gm-
16:1ω7c	Gm-, BC, Anaerobes	20:2ω6,9c	-
16:1ω8c	Gm-, Methanotrophs	20:4ω6,9,12,15c	Eukaryotic
16:1ω9c	Gm-	C20 N Alcohol	-
17:0	Bacteria: Non-specific	cis910 epoxy 18:0	-
10Me17:0	Actinomycetes	Sebacic Acid	-
*a*17:0	Gm+, BC	Sum In Feature 12–20:1ω12c	Gm-
*cy*17:0	Gm-(An), γ-proteo, BC	Sum In Feature 17–16:2ω6c	-
*i*17:0	Gm+, BC	Sum In Feature 18—*i*18:0	Gm+
17:1ω8c	Gm-, Methanotrophs	Sum In Feature 4—*i*17:1	Gm+
ɑ17:1 AT9	-	Sum In Feature 7–18:3ω3c	Eukaroyotes
*i*17:1ω9c	Gm+		

Widespread—lipid is found within many domains of life. Blank—lipid was not described as indicating a specific group in referenced reports. Abbreviations: Gram-negative (Gm-); Gram-positive (Gm+); γ-Proteobacteria (γ-proteo EV = *Enterobacter/Vibrio*); *Bacillus-Clostridium* group (BC); Actinobacteria (Act); Anaerobes (An); Saprotrophic Fungi (SP); Arbuscular mycorrhizal fungi (AMF); Ectomycorrhizal fungi (Ecto).

### Differences by Hive, Sample Year, Hive Component and Geographic Location—Univariate Analysis

Lipid abundances across all samples were summed and averaged and all those that represented greater than 1% of biomass were graphed as the main hive community members. As a basic community richness index, the absolute counts of the number of unique lipids detected in each component were averaged and presented with Standard Error of the Mean. Significant differences between each component group were explored via ANOVA followed by Tukey Kramer HSD pairwise comparisons.

Lipid accumulation curves were generated by using EstimateS v.8.2.0 [46] to calculate both observed and estimated richness with the *Mao Tau* and *Chao2* (1000 randomizations & bias corrected when applicable) functions respectively. These points were then plotted along with the estimated 95% confidence intervals forming envelopes. Components were split and presented on two different graphs to enhance legibility.

Proportional community representations for each component were generated by averaging the dataset representing each lipid and then summing these lipids according to their indicated taxa. These proportions were then presented excluding the IS (internal standard), widespread and unknown/non-specific groups in order to increase the resolution of the other, more informative, groups. ANOVA followed by Tukey Kramer HSD pairwise comparisons were then completed between components on the taxa-pooled data and individual lipid profiles. This uncovered any significant differences between components based on organismal groups and individual lipids. All statistical analyses for this study were completed with JMP (Version 9.0.2. SAS Institute Inc., Cary, NC, 1989–2012) unless otherwise noted.

All samples were grouped by hive of origin, sample year, hive component or geographic location. The resulting set of values for each individual lipid was compared between groups via one-way ANOVA. Tests resulting in significant differences were then further explored by Tukey-Kramer HSD pairwise comparisons.

### Differences by Hive, Sample Year, Hive Component and Geographic Location—Multivariate Analysis

PCA was carried out using pairwise estimates and a lipid profile matrix was constructed with each row corresponding to a specific sample and each column corresponding to a lipid detected across samples. Two-way clustering analysis was also performed with this matrix. The previous PCA indicated that outliers may be present; as such, a two-way clustering analysis employing a Centroid method more robust to outliers was applied to this matrix [47].

## Results and Discussion

Hive components from a total of 11 different *Apis mellifera* colonies were sampled from 4 different locations (S1 Table) in south central Wisconsin during the midsummers and early falls of 2006, 2008 and 2010. This resulted in 70 samples from which profiles of 59 different lipids were analyzed.

### Microbial Communities of Hives

**Fig 1:**
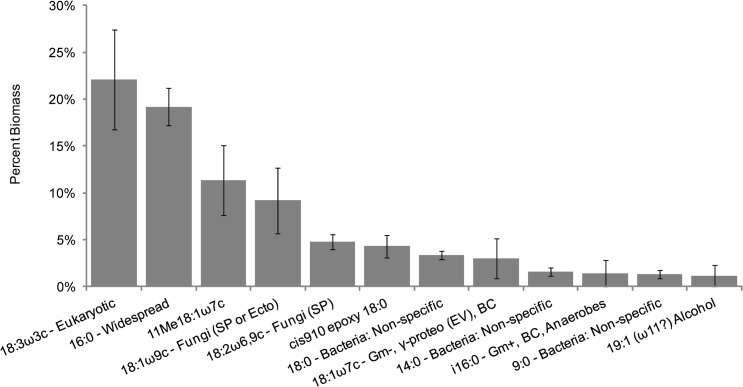
Average abundance of the most common lipids within honey bee colonies. Bar graph representing the average biomass of the most common lipids on a whole hive basis, generated by combining the individual hive components. Presented are those lipids that represent more than 1%. Error bars represent standard error of the mean. Abbreviations: Gram-negative (Gm-); Gram-positive (Gm+); γ-Proteobacteria (γ-proteo EV = *Enterobacter/Vibrio*); *Bacillus-Clostridium* group (BC); Saprotrophic Fungi (SP); Ectomycorrhizal fungi (Ecto).

A summary of the major microbial community members found throughout honey bee colonies was produced by combining all profiles by hive. This revealed 12 lipid indicators that each represent more than one percent of the detected communities (Fig 1 and S1 Fig). The profiles produced are the fractions of biomass each community member represents in each sample. Thus, when discussing abundance, a discreet number of individuals within each sample is not being indicated. The top lipid indicator 18:3ω3c (22.1%) represents general Eukaryotes, and is likely from the honey bees themselves. The second most prevalent lipid, 16:0 (19.2%), is found in phylogenetically diverse organisms and as such, this signal likely represents an aggregation of many different organisms in the hive. The fungal lipids 18:1ω9c (9.2%) and 18:2ω6,9c (4.8%) were abundant within hives, indicating that fungi are important members of honey bee colony communities. We cannot rule out pathogenic fungi in our study. However, given the abundance of fungal lipid markers across all 11 colonies, our findings likely support other culture and sequence-based studies that suggest the presence of beneficial fungi within honey bee hives [14,24,48]. We also detected an abundance of bacteria within hives, as indicated by non-specific bacterial lipids, 18:0 (3.4%), 14:0 (1.6%) and 9:0 (1.3%), and Gram-negative bacterial lipids, 18:1ω7c (3.0%) and *i*16:0 (1.4%). These findings, in combination with the low abundance of Gram-positive markers, suggest that the bacterial component of hive communities may be composed of more Gram-negative than Gram-positive bacteria. The remaining lipids that we detected in higher abundance, 19:1(ω11?)Alc (1.1%), 11Me18:1ω7c (11.3%), and cis910 epoxy 18:0 (4.3%), are not associated with any specific phylogenetic group. Samples were restricted to a 1% threshold to focus on signatures that are more likely to represent prevalent community members.

When summed, the top 12 lipid indicators represent approximately 82.7% of all detected biomass in all hive samples. In contrast, a similar study in leaf-cutting ants reveals that only 66.8% of biomass is represented by a similarly restricted set of lipid indicators [3].

### Microbial Communities Associated with Hive Components

**Fig 2:**
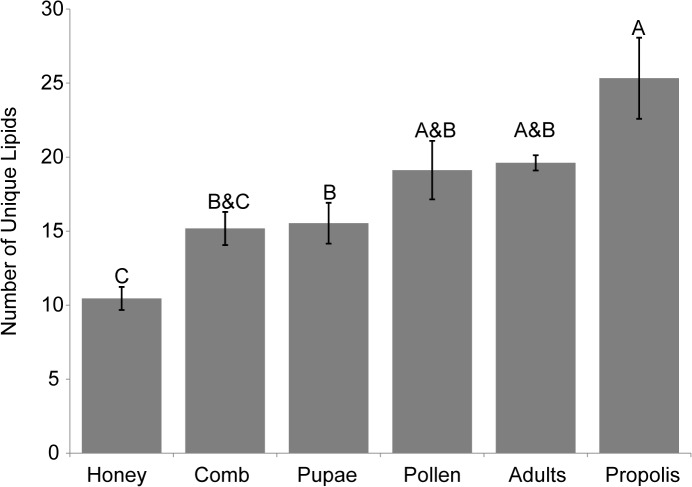
Lipid richness for honey bees and their hive components. Average number of individual lipids detected from each hive component. Error bars represent standard error of the mean. Letters represent Tukey-Kramer HSD pairwise comparisons, with those groups not possessing overlap in letters being significantly different. Those groups sharing letters do not significantly differ.

**Fig 3:**
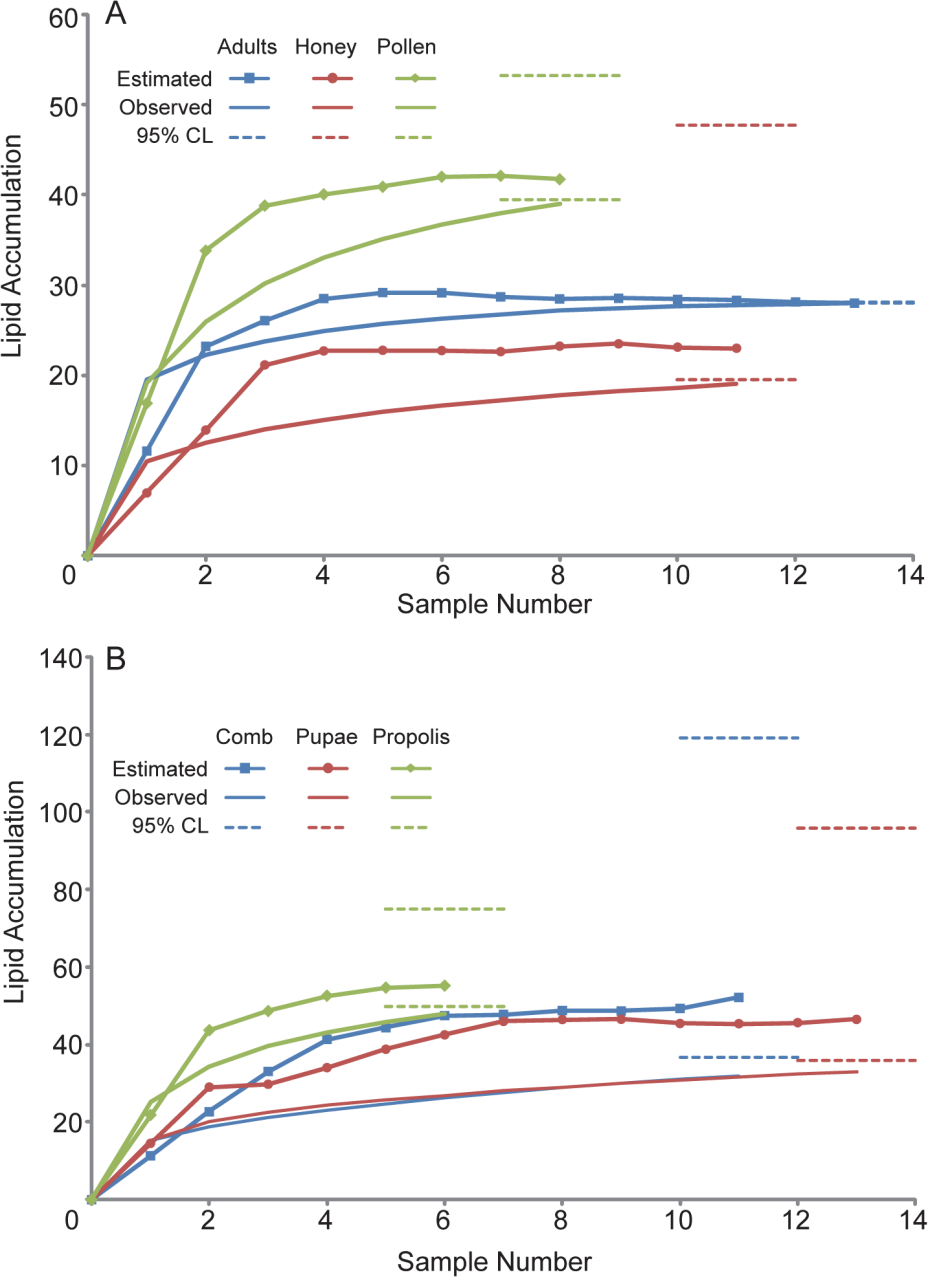
Rarefaction and lipid accumulation curves for honey bees and their hive components. **A.)** Observed and estimated curves resulting from community analyses of adults, honey, and pollen using *Mao Tau* and *Chao2* methods. **B.)** Observed and estimated curves from comb, pupae, and propolis community analyses. Dashed lines represent corresponding confidence intervals.

We detected the presence of microbially derived membrane lipid profiles from all six hive components sampled from the 11 honey bee colonies tested. We found significant differences in the number of unique lipids found in each component (Fig 2 and Table 2; one-way ANOVA, P-Value < 0.0001). Propolis had the highest average number of unique lipids (significantly more than honey, pupae and comb; P-values <0.0001, 0.0002 and 0.0002, respectively), and honey had the lowest (significantly fewer than propolis, adults, pollen and pupae; P-values <0.0001, <0.0001, 0.0006 and 0.0493, respectively) (Table 2). Accumulation curves of component lipid profiles indicated sampling was sufficient in all components with observed measurements approaching or surpassing the lower boundary of the 95% confidence interval (Fig 3). These curves provide further support for microbial diversity being highest in propolis and lowest in honey, with these two differing significantly in lipid richness.

**Table 2:** P-values from Tukey-Kramer HSD Pairwise Comparisons of Total Number of Lipids Detected in Each Component.

**Comparison**		**P-values**	**Comparison**		**P-values**
Propolis	Honey	<.0001	Comb	Honey	NS
Propolis	Comb	0.0002	Adults	Comb	NS
Propolis	Pupae	0.0002	Adults	Pupae	NS
Adults	Honey	<.0001	Pollen	Comb	NS
Pollen	Honey	0.0006	Pollen	Pupae	NS
Pupae	Honey	0.0493	Adults	Pollen	NS
Propolis	Pollen	NS	Pupae	Comb	NS
Propolis	Adults	NS			

NS—Not Significant

**Fig 4:**
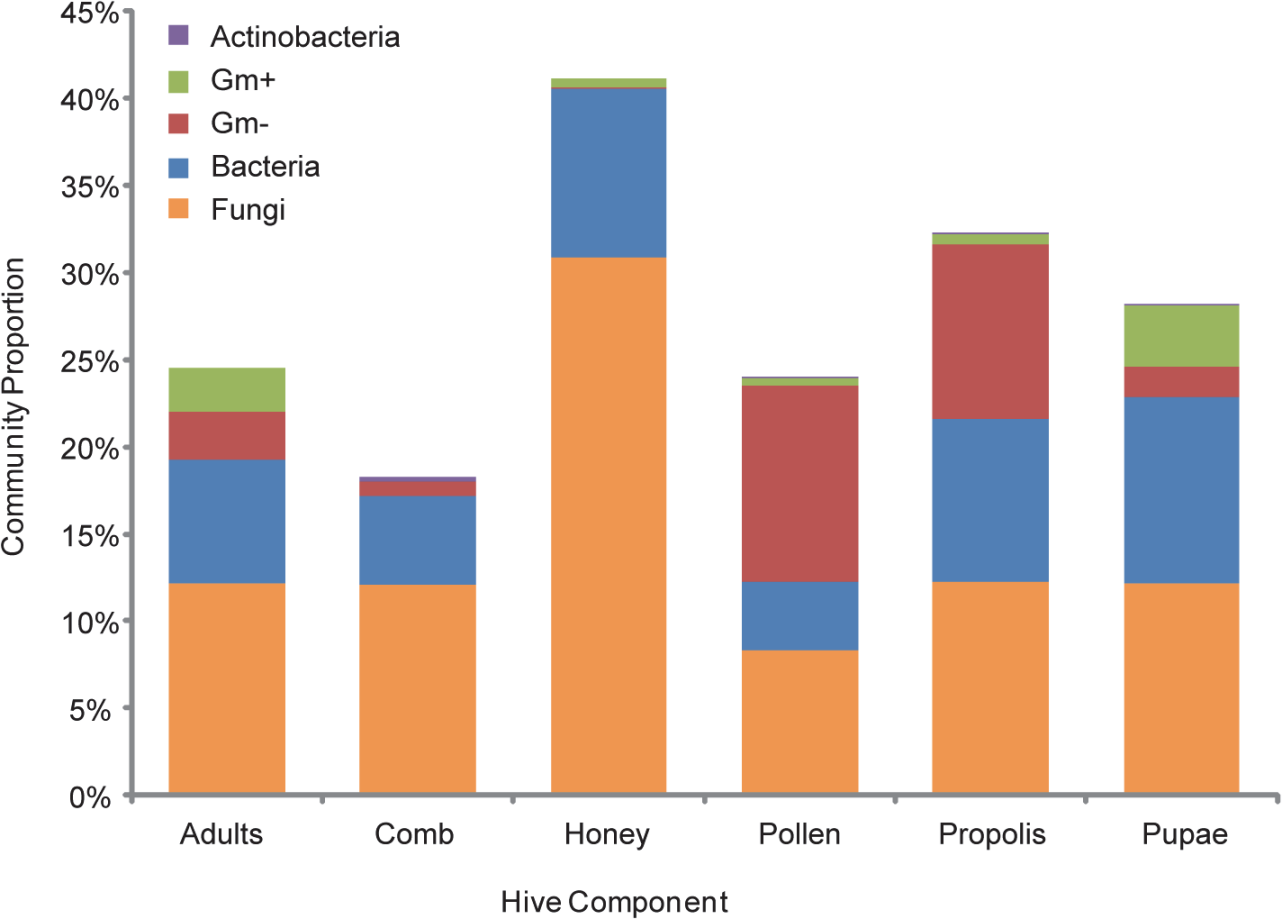
Lipid-based microbial community profile for honey bees and their hive components. Relative abundance of Actinobacteria, Gram-positive, Gram-negative, general bacteria, and fungi associated with honey bees and the different components of honey bee colonies.

Fungal markers were abundant in all components (Fig 4). The fungal proportion of communities was greatest in honey (30.9%) and lowest in pollen (8.3%), with each of the other components being represented by approximately 12%. Amplicon and metagenomic studies [17,19,49–51] have not identified an abundance of fungi associated with worker bees. This difference is likely due to these studies focusing largely on their guts, but could also reflect differences in the biases of DNA- versus lipid-based approaches. Fungi have been isolated from worker bees in culture based studies [24]. Further culture independent work is required to determine the prevalence and ecological role of fungi in the microbiome of honey bees and their hives. Bacteria were also abundant in all samples. Gram-negative bacteria represented a larger percentage of communities in comparison to Gram-positives in all components. Adults and pupae exhibited the highest proportion of Gram-positive bacteria.

Our finding of microbial indicators in adult honey bees, pupae, pollen, and comb is expected as microbes have been previously shown to be associated with these components [14,22,24,52–55]. However, their presence in honey and propolis is surprising, as both are thought to be relatively aseptic. The suppression of microbial growth in honey is thought to occur due to its high osmolarity, acidity, and the presence of antibiotic compounds [56]. This may help explain why we found the fewest microbial indicators in honey. Conversely, propolis gave rise to the largest number of unique indicators and the most diverse community profile, even though the defensive chemicals found in the tree resins from which it is made are thought to impart antibiotic characteristics [57]. This is likely, at least in part, due to propolis samples being scraped from hives and not collected before application.

### Similarity of Microbial Communities by Component, Location, Year and Hive

To begin to determine if the microbial communities within honey bee colonies are structured by hive, geographic location, sampling year, or hive component, we performed one-way ANOVAs and Tukey-Kramer HSD tests on lipid profiles that were synthesized by grouping each individual lipid by these different factors (Tables 3 and 4). Analysis by geographic location showed that only one lipid (20:1ω5c, Gram negative) differed significantly (p-value = 0.0270), with Middleton hives being distinguished from Waukesha (p-value = 0.386) and Mount Horeb (p-value = 0.0169) hives. When studied by individual hive, lipid profiles from 18:3ω6,9,12c (Eukaryotes) and C20 N Alcohol (Not defined) revealed significant differences. Additional pairwise comparisons found no significant differences in lipid profiles based on individual hive. Only 8 of the lipids were found to be significantly different by sample year, with 6 of these having specific pairwise differences (Table 3). Three of those six lipids showed one year to be significantly different from both of the other two sample years, while the other three lipids only indicated differences between two years.

**Table 3:** P-Values from Tukey-Kramer HSD Pairwise Comparisons of Lipid Profiles by Year.

**Lipid**	**ANOVA**	**Contrast**	**2006**	**2008**	**2010**
C20 N Alcohol	0.0001	2006	*	<0.0001	0.034
20:4ω6,9,12,15c	0.0361	2006	*	0.0275	NS
cis910 epoxy 18:0	0.0188	2006	*	0.015	NS
18:3ω6,9,12c	<0.0001	2008	0.0002	*	0.0073
11Me18:1ω7c	0.0017	2008	0.0129	*	0.0069
18:1ω7c	0.0312	2008	NS	*	0.0312
12:0	0.046	-			
*i* 20:0	0.047	-			

*—Comparison Not Valid;

NS—Not Significant

**Table 4:** P-Values from Significant Tukey-Kramer HSD Pairwise Comparisons of Lipid Profiles By Component.

**Lipid**	**ANOVA**	**Contrast**	**Adults**	**Pupae**	**Comb**	**Honey**	**Pollen**	**Propolis**
11:0-2OH	0.0019	Propolis	0.0019	0.0022	0.0028	0.0028	0.0036	*
12:0-3OH	0.0002	Propolis	0.0002	0.0003	0.0006	0.0004	0.0005	*
12:1ω8c	0.0006	Propolis	0.0006	0.0008	0.001	0.001	0.0014	*
14:1ω7c	<.0001	Propolis	<.0001	<.0001	<.0001	<.0001	<.0001	*
14:2 ω6c/*a*14:0	<.0001	Propolis	<.0001	<.0001	<.0001	<.0001	<.0001	*
*a*17:0	<.0001	Propolis	<.0001	<.0001	0.0002	<.0001	0.0002	*
*cy*17:0	0.0022	Propolis	0.0022	0.0026	0.0032	0.0032	0.0042	*
18:1ω6c	0.0231	Propolis	0.0196	0.0224	0.0317	0.0262	0.0314	*
20:1ω5c	0.0207	Propolis	0.0173	0.0199	0.0313	0.0233	0.0281	*
20:0	<.0001	Propolis	0.0001	0.0002	NS	0.0001	0.0013	*
20:1ω6c	0.0025	Propolis	0.0019	0.0065	NS	0.0046	0.0109	*
20:4ω6,9,12,15c	0.0009	Adults	*	0.0039	0.0057	0.0053	0.0086	0.0315
16:1ω7c	<.0001	Adults	*	<.0001	<.0001	<.0001	<.0001	<.0001
*i*19:1	<.0001	Adults	*	<.0001	<.0001	<.0001	<.0001	<.0001
18:3ω3c - 1	<.0001	Adults	*	0.0016	<.0001	<.0001	0.0108	<.0001
17:0	0.0006	Adults	*	0.0018	0.012	0.0017	0.0049	NS
16:1ω9c - 1	<.0001	Adults	*	NS	0.0006	0.0001	0.0058	0.0034
18:2ω6,9c - 1	<.0001	Adults	*	0.0013	0.0009	0.0015	NS	0.0016
16:0	0.0014	Adults	*	NS	0.0062	0.0011	NS	NS
18:0–1	0.0001	Adults	*	NS	0.0054	NS	0.0007	NS
18:1ω9c	<.0001	Honey	<.0001	0.0003	0.0007	*	<.0001	0.0014
19:0	0.0002	Honey	0.0005	0.0042	0.0012	*	0.0004	0.0078
9:0	<.0001	Honey	<.0001	0.001	<.0001	*	<.0001	0.0001
18:3ω3c - 2	<.0001	Honey	0.0108	0.0205	NS	*	0.012	NS
18:1ω5c	0.0068	Pollen	0.0149	0.0388	0.0207	0.0083	*	0.0356
18:2ω6,9c - 2	<.0001	Pollen	NS	0.0433	0.0298	0.0438	*	0.0274
18:1ω7c	0.0391	Pollen	0.0498	NS	NS	NS	*	NS
14:0	0.0003	Pupae	0.0011	*	0.0022	0.0004	0.0217	0.0445
16:1ω9c - 2	<.0001	Pupae	NS	*	0.0053	0.0012	0.0344	0.0165
18:0–2	0.0001	Pupae	NS	*	0.0227	NS	0.0035	NS
11Me18:1ω7c	0.0355	Comb	0.0353	NS	*	NS	NS	NS
12:0	0.0249	-						
15:0	0.0419	-						
cis910 epoxy 18:0	0.0291	-						

*—Comparison Not Valid;

NS—Not Significant

The greatest number of individual statistical differences was observed from grouping lipid profiles by hive component (Table 4). Specifically, 34 lipid profiles exhibited significant differences among hive components, and 31 of these had specific pairwise component differences. Propolis appears to have the most unique microbial community, with nine lipids being significantly different when compared to all other components. The second most unique microbial community was observed in adult samples, which were indicated as significantly different from all other components by four lipids. Honey had 3 similarly representative lipids, and pollen and pupae each had one representative lipid. Several other pairwise lipid profile differences by hive components were also observed, where two or more components had significant differences from the other components (Table 4).

**Fig 5:**
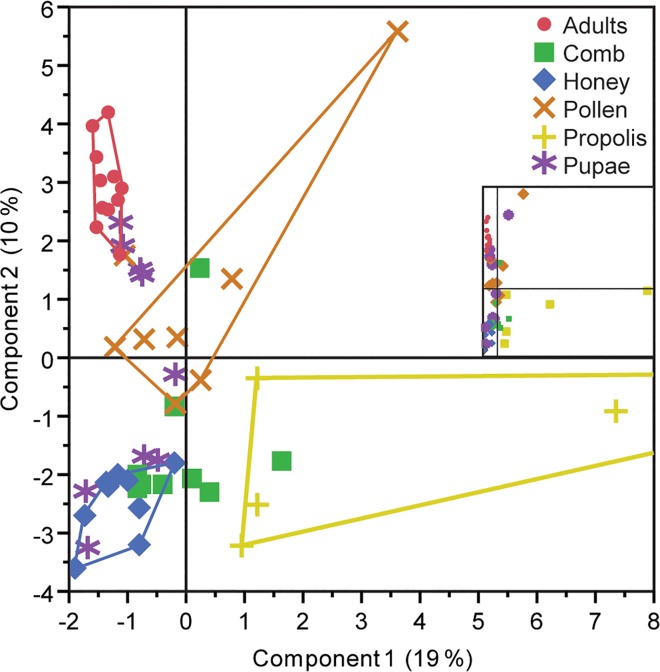
Principal component analysis of lipid profile for honey bees and their hive components. Score plot shows variation in PLFA-FAME profiles by hive components. Lines drawn delineate sample aggregates. Inlay displays one propolis sample that skewed the plot. Main plot is redrawn without that data point. When produced by sample year, individual hive or location, no clustering is readily apparent.

To further test the hypothesis that microbial communities are structured by hive component, comparisons were made of lipid profiles across our sampling variables using Principal Component Analysis (PCA). When points are labeled by year, hive or location, no clustering is apparent (data not shown). However, when comparing hive components with each other, adults, pollen, propolis and honey can be observed as distinct and non-overlapping (Fig 5). Comb and pupae overlap all other components except adults and propolis, respectively.

**Fig 6:**
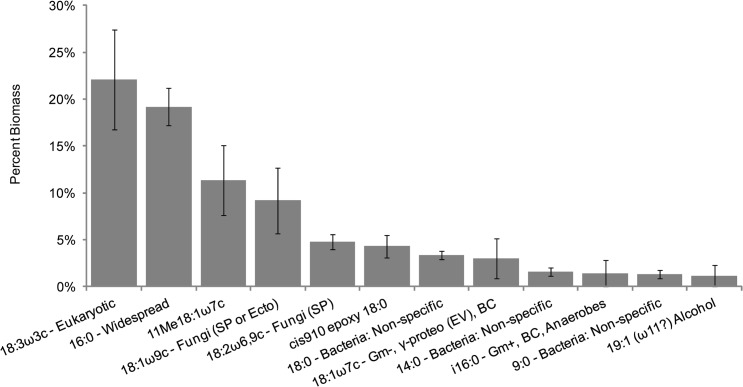
Heat map, dendrograms and dot plot representing both lipid and component clustering. Heatmap represents a two-way clustering analysis derived from lipid profiles. Vertical dendrogram represents clustering of profiles by components. This dendrogram shows components clustering together at various points in the tree. The dot plot immediately to the right of the vertical diagram represents each of the eleven hives sampled and indicates that communities are not clustering by hive as dots are not found in groups on the same row. Each of the sample labels are color coded to represent sampling year. Similar to component-wise consideration, there appear to be clusters by year, although to a lesser extent. The horizontal dendrogram at the top of the figure represents individual lipids. Clustering can also be observed when considering general lipid division, e.g. the monounsaturated fatty acids group together. Fatty acid coloring: hydroxyl, green; cyclic, orange; branched, purple; monounsaturated, red; saturated, blue; methylated, yellow; polyunsaturated, pink; unclassified, black.

To further assess structuring of microbial communities in association with honey bee hives, a two-way clustering analysis was performed using all detected lipid profiles and visualized in the form of a heat map with corresponding dendrograms and a dot plot (Fig 6). Each hive component was further color coded to indicate sampling year and a dot plot was added to represent individual hives. The dot plot (Fig 6) reveals that samples do not cluster by hive, which would be represented by dots grouping together on the same rows. There is some grouping by sampling year, but most clusters include a mix of years. Structuring by hive component is most supported as distinct clusters of each component type are distributed along the y-axis of the dendrogram and heat map (Fig 6). The corresponding clustering history (S2 Table) provides further support in components joining clusters of the same component type as within cluster distance is increased. Along with the corresponding dendrogram, the clustered set of detected lipids is displayed along the upper x-axis. The mono-unsaturated lipids, which generally indicate Gram-negative bacteria, tend to group together, while the branched chain fatty acids, which generally indicate Gram-positive bacteria, cluster together. Although, less pronounced than in component type, the clustering history (S3 Table) for detected lipids also reveals cluster joining of similar lipids at lesser distances.

Our findings strongly suggest that there are distinct microbial communities in hives and that they are partitioned by component. Specifically, three lines of evidence support this observation. First, when individual lipid profiles were compared, we found a significant number of differences between hive components, and few differences specific to year of collection, individual hive, or geographic location. Second, PCA only delineates non-overlapping groups based on hive components. Finally, a two-way cluster analysis heat map clearly demonstrated more groups of samples by component than year, geographic location, or hive.

Some of our findings may be explained by honey bee life history. For example, year-based lipid profile comparisons giving rise to 8 differing lipid profiles are most likely the result of the specific point in the active season at which samples were harvested, since these hives were sampled at various times from midsummer to early fall. As such, corresponding hive activities and available pollen and nectar sources most likely varied between sampling years. Also, in the PCA, pupae and combs overlapped several other components. This might be expected since the comb is the physical structure in which most components are found and as such, comb samples likely contained residues from other components. Furthermore, pupae actively develop into adults while sequestered in a comb cell with pollen and honey-based nutritional resources, explaining why they might overlap more with other hive components.

## Conclusion

Here, we present the first PLFA and FAME characterization of microbial communities associated with honey bees and their hives. This membrane lipid based method is advantageous as it detects viable microbes. As such, our findings provide support for the existence of a metabolically active and broadly diverse community of microbes associated with honey bees and their different hive components, including honey and propolis. Further, our study shows that the microbial community appears to be structured by different hive components of honey bee hives. These components are physically separated, partially temperature regulated, and comprised of nutritionally diverse materials. This suggests that the microbiome of honey bee colonies is complex and that generating a better understanding of all resident microbial communities of the hive could be important to understanding overall hive health [3]. Future research, building on the lipid-based study presented here and on recent amplicon and metagenomic studies of others [17,19,49–51], will further our understanding of the microbiome of honey bees and their hives.

## Supporting Information

S1 Fig Bar graph of most common lipids in each hive component. All lipids representing more than 1% biomass averaged together by hive component. Error bars represent standard error of the mean.
(DOCX)

S1 Table Sample inventory. (DOCX)

S2 Table Clustering history of hive components. (DOCX)

S3 Table Clustering history of lipid indicators. (DOCX)
