# Correction: Unique Honey Bee (*Apis mellifera*) Hive Component-Based Communities as Detected by a Hybrid of Phospholipid Fatty-Acid and Fatty-Acid Methyl Ester Analyses

**DOI:** 10.1371/journal.pone.0133100

**Published:** 2015-07-14

**Authors:** Kirk J. Grubbs, Jarrod J. Scott, Kevin J. Budsberg, Harry Read, Teri C. Balser, Cameron R. Currie

The image for Fig 6 is incorrect. Please see the corrected Fig 6 here.

**Fig 6:**
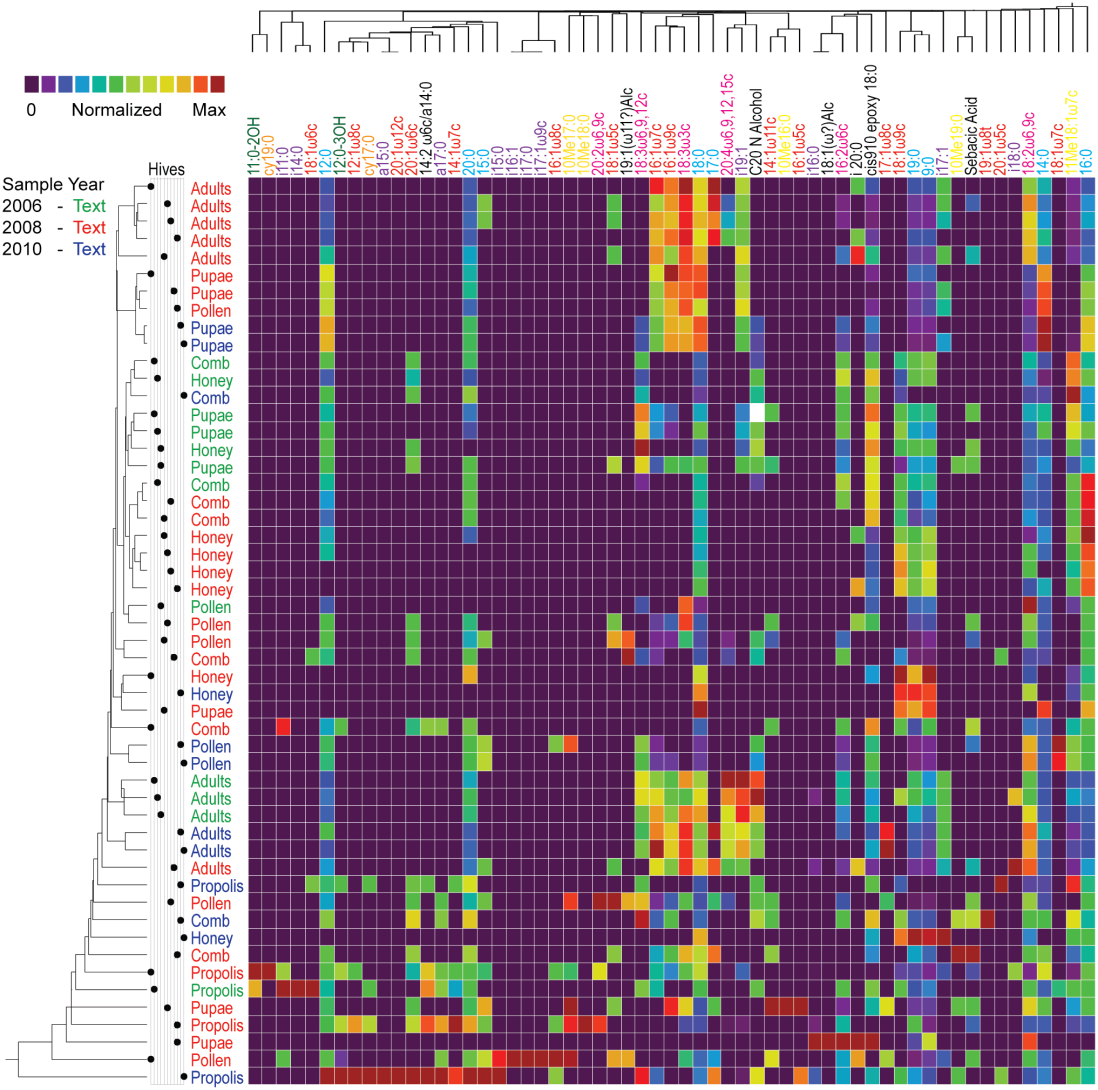
Heat map, dendrograms and dot plot representing both lipid and component clustering. Heatmap represents a two-way clustering analysis derived from lipid profiles. Vertical dendrogram represents clustering of profiles by components. This dendrogram shows components clustering together at various points in the tree. The dot plot immediately to the right of the vertical diagram represents each of the eleven hives sampled and indicates that communities are not clustering by hive as dots are not found in groups on the same row. Each of the sample labels are color coded to represent sampling year. Similar to component-wise consideration, there appear to be clusters by year, although to a lesser extent. The horizontal dendrogram at the top of the figure represents individual lipids. Clustering can also be observed when considering general lipid division, e.g. the monounsaturated fatty acids group together. Fatty acid coloring: hydroxyl, green; cyclic, orange; branched, purple; monounsaturated, red; saturated, blue; methylated, yellow; polyunsaturated, pink; unclassified, black.

## References

[1] Grubbs KJ, Scott JJ, Budsberg KJ, Read H, Balser TC, Currie CR (2015) Unique Honey Bee (*Apis mellifera*) Hive Component-Based Communities as Detected by a Hybrid of Phospholipid Fatty-Acid and Fatty-Acid Methyl Ester Analyses. PLoS ONE 10(4): e0121697 doi: 10.1371/journal.pone.0121697 25849080 10.1371/journal.pone.0121697 PMC4388481

