# Supplementary material for: Unique Honey Bee (Apis mellifera) Hive Component-Based Communities as Detected by a Hybrid of Phospholipid Fatty-Acid and Fatty-Acid Methyl Ester Analyses
Source: PLoS One. 2015 Apr 7;10(4):e0121697. doi: 10.1371/journal.pone.0121697 (PMC4388481; doi:10.1371/journal.pone.0121697)
Supplement: S1 Table — (DOCX) [file pone.0121697.s002.docx]

| **Hive Component** | **Hive Location** | **Year** | **Mass (G)** | **Hive Component** | **Hive Location** | **Year** | **Mass (G)** |
| --- | --- | --- | --- | --- | --- | --- | --- |
| Pupae | Mount Horeb | 2006 | 1 | Comb | Mount Horeb | 2008 | 0.913 |
| Pupae | Mount Horeb | 2006 | 1 | Honey | Mount Horeb | 2008 | 1.483 |
| Comb | Mount Horeb | 2006 | 1 | Pollen | Madison | 2008 | 1.1 |
| Comb | Mount Horeb | 2006 | 1 | Adults | Madison | 2008 | 1.293 |
| Honey Comb | Mount Horeb | 2006 | 1 | Pupae | Madison | 2008 | 1.026 |
| Honey Comb | Mount Horeb | 2006 | 1 | Comb | Madison | 2008 | 0.04895 |
| Propolis | Mount Horeb | 2006 | 1 | Honey | Madison | 2008 | 0.828 |
| Propolis | Mount Horeb | 2006 | 1 | Adults | Waukesha | 2008 | 0.1115 |
| Adults | Mount Horeb | 2006 | 1 | Pupae | Waukesha | 2008 | 1.089 |
| Pupae | Mount Horeb | 2006 | 1 | Pollen | Waukesha | 2008 | 0.115 |
| Pupae | Mount Horeb | 2006 | 1 | Honey | Waukesha | 2008 | 0.829 |
| Larvae | Mount Horeb | 2006 | 1 | Adults | Waukesha | 2008 | 0.902 |
| Comb | Mount Horeb | 2006 | 1 | Honey | Waukesha | 2008 | 1.185 |
| Comb | Mount Horeb | 2006 | 1 | Comb | Waukesha | 2008 | 0.844 |
| Honey | Mount Horeb | 2006 | 1 | Pollen | Waukesha | 2008 | 1.273 |
| Honey | Mount Horeb | 2006 | 1 | Comb | Waukesha | 2008 | 1 |
| Adults | Mount Horeb | 2006 | 1 | Adults | Waukesha | 2008 | 0.969 |
| Adults | Mount Horeb | 2006 | 1 | Honey | Waukesha | 2008 | 1.565 |
| Pupae | Mount Horeb | 2006 | 1 | Pupae | Waukesha | 2008 | 1.122 |
| Pupae | Mount Horeb | 2006 | 1 | Pupae | Madison | 2010 | 0.269 |
| Honey | Mount Horeb | 2006 | 1 | Propolis | Madison | 2010 | 0.258 |
| Honey | Mount Horeb | 2006 | 1 | Adults | Madison | 2010 | 0.256 |
| Adults | Mount Horeb | 2006 | 1 | Adults | Madison | 2010 | 0.256 |
| Adults | Mount Horeb | 2006 | 1 | Honey | Madison | 2010 | 0.33 |
| Pollen | Mount Horeb | 2006 | 1 | Pollen | Madison | 2010 | 0.285 |
| Adults | Mount Horeb | 2008 | 0.1031 | Pollen | Madison | 2010 | 0.285 |
| Pupae | Mount Horeb | 2008 | 0.236 | Comb | Madison | 2010 | 0.251 |
| Propolis | Mount Horeb | 2008 | 1.065 | Comb | Madison | 2010 | 0.251 |
| Pollen | Mount Horeb | 2008 | 0.918 | Pupae | Middleton | 2010 | 0.222 |
| Comb | Mount Horeb | 2008 | 0.972 | Propolis | Middleton | 2010 | 0.245 |
| Honey | Mount Horeb | 2008 | 0.911 | Propolis | Middleton | 2010 | 0.245 |
| Adults | Mount Horeb | 2008 | 0.191 | Adults | Middleton | 2010 | 0.261 |
| Pupae | Mount Horeb | 2008 | 0.917 | Honey | Middleton | 2010 | 0.221 |
| Propolis | Mount Horeb | 2008 | 1.088 | Pollen | Middleton | 2010 | 0.321 |
| Pollen | Mount Horeb | 2008 | 1.059 | Comb | Middleton | 2010 | 0.239 |

**S1 Table. Sample inventory.**

Mass (G) refers to the mass of sample analyzed.
