# Supplementary material for: Unique Honey Bee (Apis mellifera) Hive Component-Based Communities as Detected by a Hybrid of Phospholipid Fatty-Acid and Fatty-Acid Methyl Ester Analyses
Source: PLoS One. 2015 Apr 7;10(4):e0121697. doi: 10.1371/journal.pone.0121697 (PMC4388481; doi:10.1371/journal.pone.0121697)
Supplement: S2 Table — (DOCX) [file pone.0121697.s003.docx]

| Number of Clusters | Distance | Leader | Joiner |
| --- | --- | --- | --- |
| 51 | 0.456626 | Pupae | Pupae |
| 50 | 0.785319 | Comb | Comb |
| 49 | 1.104479 | Honey | Honey |
| 48 | 1.274043 | Pupae | Pollen |
| 47 | 1.5719 | Adults | Adults |
| 46 | 1.639764 | Comb | Comb |
| 45 | 1.744454 | Pupae | Pupae |
| 44 | 1.789686 | Adults | Adults |
| 43 | 1.939042 | Pupae | Pupae |
| 42 | 2.108496 | Honey | Honey |
| 41 | 2.173511 | Comb | Honey |
| 40 | 2.194601 | Adults | Adults |
| 39 | 2.262825 | Pupae | Pupae |
| 38 | 2.483433 | Honey | Honey |
| 37 | 2.713834 | Pupae | Honey |
| 36 | 2.774692 | Comb | Comb |
| 35 | 3.076266 | Comb | Honey |
| 34 | 3.150341 | Pollen | Pollen |
| 33 | 3.172493 | Pollen | Pollen |
| 32 | 3.271723 | Pupae | Pupae |
| 31 | 3.626858 | Comb | Pupae |
| 30 | 3.717703 | Comb | Comb |
| 29 | 3.744483 | Adults | Adults |
| 28 | 3.991081 | Pollen | Comb |
| 27 | 4.031616 | Comb | Pollen |
| 26 | 4.139595 | Honey | Honey |
| 25 | 4.404265 | Honey | Pupae |
| 24 | 4.421952 | Adults | Adults |
| 23 | 3.949534 | Adults | Adults |
| 22 | 4.944229 | Adults | Adults |
| 21 | 5.316306 | Adults | Pupae |
| 20 | 5.152559 | Adults | Comb |
| 19 | 5.420399 | Adults | Pollen |
| 18 | 5.864105 | Adults | Honey |
| 17 | 6.19287 | Adults | Comb |
| 16 | 6.361985 | Adults | Pollen |
| 15 | 6.774294 | Adults | Adults |
| 14 | 6.884483 | Adults | Adults |
| 13 | 7.803711 | Adults | Adults |
| 12 | 8.577099 | Adults | Propolis |
| 11 | 9.272417 | Adults | Pollen |
| 10 | 9.435224 | Adults | Comb |
| 9 | 9.75919 | Adults | Honey |
| 8 | 10.1487 | Adults | Comb |
| 7 | 10.78307 | Adults | Propolis |
| 6 | 12.39389 | Adults | Propolis |
| 5 | 13.13084 | Adults | Pupae |
| 4 | 13.12558 | Adults | Propolis |
| 3 | 14.03926 | Adults | Pupae |
| 2 | 16.52377 | Adults | Pollen |
| 1 | 22.34226 | Adults | Propolis |

**S2 Table. Clustering history of hive components.**
