# Supplementary material for: Unique Honey Bee (Apis mellifera) Hive Component-Based Communities as Detected by a Hybrid of Phospholipid Fatty-Acid and Fatty-Acid Methyl Ester Analyses
Source: PLoS One. 2015 Apr 7;10(4):e0121697. doi: 10.1371/journal.pone.0121697 (PMC4388481; doi:10.1371/journal.pone.0121697)
Supplement: S3 Table — (DOCX) [file pone.0121697.s004.docx]

| Number of Clusters | Distance | Leader | Joiner |
| --- | --- | --- | --- |
| 58 | 0 | *a*15:0 | Sum In Feature 12 - 20:1ω12c |
| 57 | 7.17E-15 | *i*17:0 | ɑ17:1 AT9 |
| 56 | 1.98E-14 | *i*16:1 | *i*17:0 |
| 55 | 0.066119 | *i*16:0 | 18:1(ω ?) Alc |
| 54 | 1.152951 | *i*14:0 | 18:1ω6c |
| 53 | 1.774834 | 12:1 ω8c | *cy*17:0 |
| 52 | 1.814131 | 12:0-3OH | 12:1ω8c |
| 51 | 2.029896 | *i*16:1 | 16:1ω8c |
| 50 | 2.483344 | 12:0-3OH | *a*15:0 |
| 49 | 2.717158 | 14:1ω11c | 10Me16:0 |
| 48 | 2.718036 | 10Me19:0 | Sebacic Acid |
| 47 | 3.022158 | 14:2ω6c/*a*14:0 | *a*17:0 |
| 46 | 3.058443 | 19:0 | 9:0 |
| 45 | 3.106143 | 11:0-2OH | *cy*19:0 |
| 44 | 3.273436 | *i*16:0 | Sum In Feature 17 - 16:2ω6c |
| 43 | 3.428633 | 12:0-3OH | 20:1ω6c |
| 42 | 3.546648 | 12:0-3OH | 14:2ω6c/*a*14:0 |
| 41 | 3.684052 | 18:1ω9c | 19:0 |
| 40 | 4.098119 | 14:1ω11c | 16:1ω5c |
| 39 | 4.281215 | 20:4ω6,9,12,15c | *i*19:1 |
| 38 | 4.341906 | 12:0-3OH | 14:1ω7c |
| 37 | 4.458666 | 16:1ω9c | Sum In Feature 7 - 18:3ω3c |
| 36 | 4.49803 | 12:0-3OH | 20:0 |
| 35 | 4.624117 | 16:1ω7c | 16:1ω9c |
| 34 | 4.863824 | 20:4ω6,9,12,15c | C20 N Alcohol |
| 33 | 4.911558 | 12:0-3OH | 15:0 |
| 32 | 5.29066 | 12:0-3OH | *i*15:0 |
| 31 | 5.291266 | *i*11:0 | *i*14:0 |
| 30 | 5.469442 | 12:0 | 12:0-3OH |
| 29 | 6.318789 | 16:1ω7c | 18:0 |
| 28 | 6.383854 | *i*16:0 | *i*20:0 |
| 27 | 6.472563 | 10Me18:0 | 20:2ω69c |
| 26 | 6.46176 | 10Me17:0 | 10Me18:0 |
| 25 | 6.6768 | 16:1ω7c | 17:0 |
| 24 | 6.713089 | *i*16:0 | cis 910 epoxy 18:0 |
| 23 | 6.798435 | 18:1ω5c | 19:1(ω11?)Alc |
| 22 | 7.050146 | 10Me17:0 | 18:1ω5c |
| 21 | 7.078951 | 16:1ω7c | 20:4ω6,9,12,15c |
| 20 | 7.130696 | *i*16:1 | 10Me17:0 |
| 19 | 7.17984 | 12:0 | *i*16:1 |
| 18 | 7.189481 | 12:0 | 18:3ω6,9,12c |
| 17 | 7.135047 | 12:0 | 16:1ω7c |
| 16 | 6.9212 | 12:0 | 14:1ω11c |
| 15 | 7.222675 | 18:1ω9c | Sum In Feature 4 - i17:1 |
| 14 | 7.264554 | 12:0 | *i*16:0 |
| 13 | 7.23681 | 12:0 | 17:1ω8c |
| 12 | 7.311682 | *i*11:0 | 12:0 |
| 11 | 7.306043 | *i*11:0 | 18:1ω9c |
| 10 | 7.289159 | 11:0-2OH | *i*11:0 |
| 9 | 7.312582 | 11:0-2OH | 10Me18:0 |
| 8 | 7.362448 | 11:0-2OH | 19:1ω8t |
| 7 | 7.372737 | 11:0-2OH | 20:1ω5c |
| 6 | 7.40118 | 11:0-2OH | Sum In Feature 18 - i18:0 |
| 5 | 7.412115 | 11:0-2OH | 18:2ω6,9c |
| 4 | 7.59632 | 11:0-2OH | 14:0 |
| 3 | 7.651591 | 11:0-2OH | 18:1ω7c |
| 2 | 7.737492 | 11:0-2OH | 11Me18:1ω7c |
| 1 | 8.059986 | 11:0-2OH | 16:0 |

**S3 Table. Clustering history or lipid indicators**
