# Supplementary material for: Unique Honey Bee (Apis mellifera) Hive Component-Based Communities as Detected by a Hybrid of Phospholipid Fatty-Acid and Fatty-Acid Methyl Ester Analyses
Source: PLoS One. 2015 Apr 7;10(4):e0121697. doi: 10.1371/journal.pone.0121697 (PMC4388481; doi:10.1371/journal.pone.0121697)
Supplement: Supplementary file 4 [file pone.0121697.s001.docx]

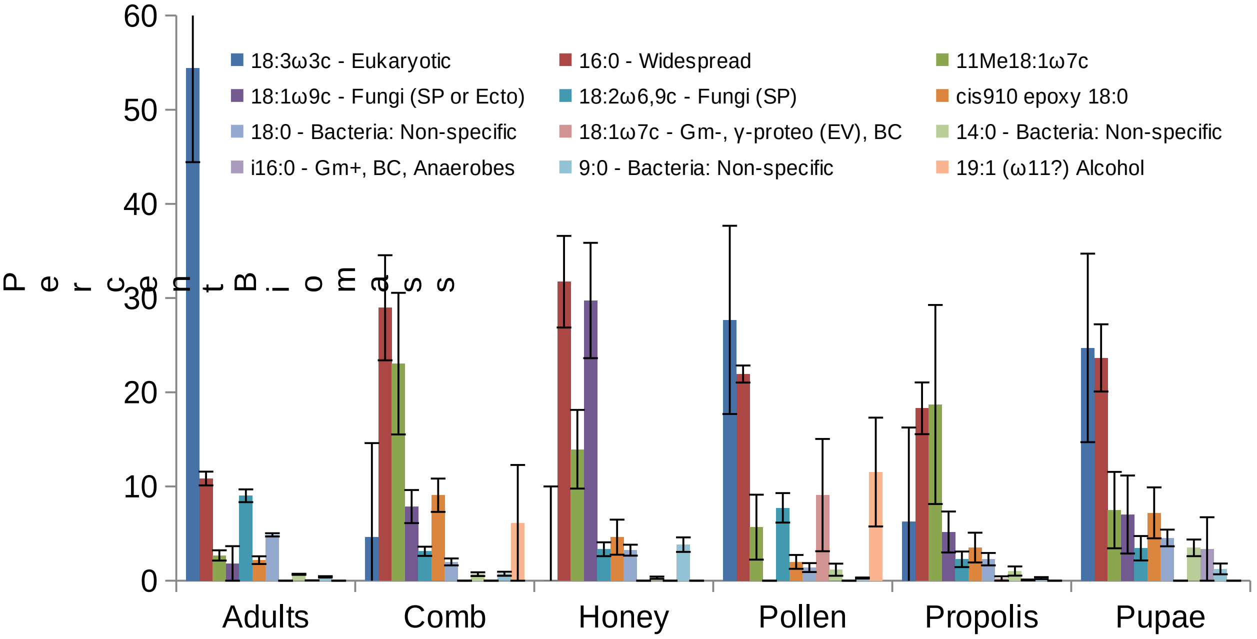

Percent Biomass
60
50
40
30
20
10
0
18:3ω3c - Eukaryotic
16:0 - Widespread
11Me18:1ω7c
18:1ω9c - Fungi (SP or Ecto)
18:2ω6,9c - Fungi (SP)
cis910 epoxy 18:0
18:0 - Bacteria: Non-specific
18:1ω7c - Gm-, γ-proteo (EV), BC
14:0 - Bacteria: Non-specific
i16:0 - Gm+, BC, Anaerobes
9:0 - Bacteria: Non-specific
19:1 (ω11?) Alcohol
Adults
Comb
Honey
Pollen
Propolis
Pupae
